# Change in Physical and Mental Quality-of-Life between the Short- and Mid-Term Periods after Cervical Laminoplasty for Cervical Spondylotic Myelopathy: A Retrospective Cohort Study with Minimum 5 Years Follow-up

**DOI:** 10.3390/jcm11175230

**Published:** 2022-09-04

**Authors:** Koji Tamai, Akinobu Suzuki, Hidetomi Terai, Minori Kato, Hiromitsu Toyoda, Shinji Takahashi, Akito Yabu, Yuta Sawada, Masayoshi Iwamae, Hiroaki Nakamura

**Affiliations:** Department of Orthopedics, Osaka Metropolitan University Graduate School of Medicine, Osaka 545-8585, Japan

**Keywords:** well-being, quality-of-life, laminoplasty, cervical spondylotic myelopathy, spinal surgery, mid-term, improvement, deterioration, outcome, decompression

## Abstract

The mid-term surgical outcomes of cervical spondylotic myelopathy (CSM), evaluated using the cervical Japanese Orthopedic Association (cJOA) score, are reported to be satisfactory. However, there remains room for improvement in quality-of-life (QOL), especially after short-term follow-up. We aimed to demonstrate changes in mental and physical QOL between short- and mid-term follow-ups and determine the predictive factors for deterioration of QOL. In this retrospective cohort study, 80 consecutive patients underwent laminoplasty for CSM. The outcome measures were Short Form-36 Physical Component Summary (PCS), Mental Component Summary (MCS), and cJOA scores. PCS and MCS scores were compared at the 2- and 5-year postoperative time points. Additionally, a multivariate logistic regression model was used to identify the predictive factors for deterioration. Significant factors in the logistic regression analysis were analyzed using receiver-operating characteristic curves. The results showed that MCS scores did not deteriorate after 2 years postoperatively (*p* = 0.912). Meanwhile, PCS significantly declined between 2 and 5 years postoperatively (*p* = 0.008). cJOA scores at 2 years postoperatively were significantly associated with PCS deterioration at 2-year follow-up. In conclusion, only physical QOL might show deterioration after short-term follow-up. Such deterioration is likely in patients with a cJOA score <13.0 at 2 years postoperatively.

## 1. Introduction

Well-being or quality-of-life (QOL) is an essential and fundamental concept of human life [1]. In general, health-related QOL can be classified into physical and mental QOL [2,3]. Spine-related disabilities, including neurological symptoms, pain, and numbness greatly impair daily life activities, resulting in a significant impairment not only in physical QOL but also in mental QOL [4,5].

Cervical spondylotic myelopathy (CSM) is the most frequent abnormality of the cervical spine, with an incidence of ≥605 per million in the North American region [6]. Hence, surgical decompression, such as laminoplasty, is the gold-standard procedure for preventing the progression of neurological deficits in patients with CSM [7]. The current treatment trends in the United States show that laminoplasty is the third-most commonly performed procedure [8].

In the spine field, outcomes are defined as short-term outcomes (at 2 postoperative years), mid-term outcomes (at 5 years), and long-term outcomes (at >10 years) [9,10]. The mid- to long-term surgical outcomes of laminoplasty, evaluated using the cervical Japanese Orthopedic Association (cJOA) score, have been reportedly satisfactory [11,12,13,14]. The cJOA score is a physician-oriented score used to assess the severity of myelopathy rather than assess the patient’s QOL. Although several studies have demonstrated the change in QOL after surgery for CSM, most only reported short-term follow-up findings [7,15]. Additionally, no study has reported the predictive factors at the short-term follow-up for the change in QOL afterwards, which could significantly assist physicians in identifying patients who require long-term follow-up. Therefore, the current study aimed to demonstrate the change in mental and physical QOL between short-term and mid-term follow-ups. Additionally, we aimed to determine the predictive factors at short-term follow-up for the deterioration of patient QOL after short-term observation.

## 2. Materials and Methods

### 2.1. Study Design and Ethics

This retrospective cohort study included patients who underwent cervical decompression surgery for CSM. All methods were performed in accordance with the Declaration of Helsinki and ethical guidelines for medical and health research involving human subjects in Japan. All study participants provided informed consent, and the study protocol was approved by the institutional review board of our institution (No. 3170).

### 2.2. Patient Population

To eliminate inconsistency in the surgical method and the effect of missing data, we selected 80 consecutive patients who underwent laminoplasty for CSM at our institution between 2009 and 2014, were followed-up for at least 5 years, and had complete postoperative 3-month, 2-year, and 5-year clinical data. Patients with a history of cervical surgery, cervical ossification of posterior longitudinal ligament, and/or those aged <30 years were excluded.

### 2.3. Surgical Procedure

All patients underwent open-door laminoplasty [16]. The surgical indication and approach were considered on a case-by-case basis by the attending physician. Hydroxyapatite spacers or anchor screws were used at each level for fixation of the opened laminae [17]. The day after surgery, all patients were provided a soft cervical collar and allowed to sit up and, if possible, to stand and walk. Collar removal was allowed 1 week after surgery. All patients were then encouraged to start range-of-motion and isometric strengthening exercises of the neck as early as possible.

### 2.4. Radiological Evaluations

The severity of preoperative spinal canal stenosis was evaluated using a previously reported classification based on preoperative magnetic resonance imaging (MRI) findings. The levels that showed grade 2 or 3 stenosis were defined as stenosis levels, and the number of stenosis levels was counted in each patient [18]. Additionally, a snake-eye appearance was defined as a lateral or bilateral small, round, high-signal-intensity lesion in the central gray matter near the ventrolateral posterior column [19].

### 2.5. Clinical Evaluations

Data on postoperative complications, including surgical site infection, C5 nerve root palsy, and reoperation rates, were collected. C5 palsy was defined as deterioration in muscle power by at least 1 grade in the manual motor test, and surgical site infection was defined as a deep infection that required additional surgery. The cJOA score was evaluated preoperatively and at 3 months, 2 years, and 5 years postoperatively [20]. Patient-oriented questionnaire scores, including the visual analog scale (VAS) and the 36-item Short Form Health Survey (SF-36) scores, were obtained preoperatively and at 3 months, 2 years, and 5 years postoperatively [21,22]. The VAS scores of neck pain, arm pain, and arm numbness were evaluated using a 10 cm long horizontal line with extremes indicated as “no symptoms” and “worst symptoms imaginable.” The SF-36 is a 36-item scale measuring eight domains of health-related QOL: physical functioning (PF), physical role limitations (RP), bodily pain (BP), general health perceptions (GH), energy/vitality (VT), social functioning (SF), emotional role limitations (RE), and mental health (MH). The domain scores were summarized as the physical component summary (PCS) and mental component summary (MCS) scores using a previously proposed algorithm [22].

### 2.6. QOL Parameters

The PCS and MCS scores of the SF-36 were used as parameters of physical and mental QOL, respectively [23]. The minimum clinically important difference for both PCS and MCS scores was reported as 4.0. Hence, the “deterioration” of PCS and MCS was defined as a decrease of >4.0.

### 2.7. Study Design & Statistical Analysis

All analyses were performed using the SPSS software (version 23; SPSS, Chicago, IL, USA). Statistical significance was set at *p* < 0.05.

#### 2.7.1. Main Analysis

The main analysis was performed to identify the change in PCS and MCS scores between 2 and 5 years postoperatively. The average change in SF-36 PCS and MCS scores obtained preoperatively and 3 months, 2 years, and 5 years postoperatively was evaluated using one-way repeated measures analysis of variance (ANOVA) with the calculation of the overall *p*-value. In subsequent post-hoc analyses, *t*-tests with Bonferroni correction were performed to evaluate differences between the scores at 2 and 5 years postoperatively. Using the chi-squared test, we also compared the number of patients with deterioration between 2 and 5 years postoperatively according to PCS and MCS scores.

#### 2.7.2. Sub-Analysis

Sub-analyses were performed only for parameters that showed significant deterioration between 2 and 5 years postoperatively in the main analysis to identify the predictive factors for the deterioration. Univariate comparison of the change in the scores of the SF-36 domains between 2 and 5 years postoperatively in patients with and without deterioration was performed using the Mann–Whitney U test. Additionally, a multivariate logistic regression model was used to identify the predictive factors for deterioration 2 years postoperatively. The explanatory variables were age, sex, and cJOA and PCS/MCS scores at 2 years postoperatively, and the objective variable was patients with deterioration. The adjusted odds ratios (aORs) and 95% confidence intervals (CIs) of the explanatory variables were calculated. Finally, significant factors in the logistic regression analysis were separately analyzed using receiver-operating characteristic (ROC) curves to validate the results and determine the cutoff value to predict the deterioration. The area under the ROC curve (AUC) and 95% CIs were calculated. The cutoff value was determined by measuring the distance from the top-left corner of the ROC curve.

## 3. Results

### 3.1. Patient Details

The patient demographics are presented in Table 1. The average age of patients (25 female and 55 male patients) was 64.0 ± 11.2 years. The cJOA score and VAS score of neck pain, arm pain, and arm numbness significantly improved after surgery (cJOA: *p* < 0.001, neck pain: *p* = 0.018, arm pain: *p* = 0.045, and arm numbness: *p* < 0.001; one-way repeated measures ANOVA).

### 3.2. Main Analysis

#### 3.2.1. Average Change in PCS and MCS Scores

**Figure 1:**
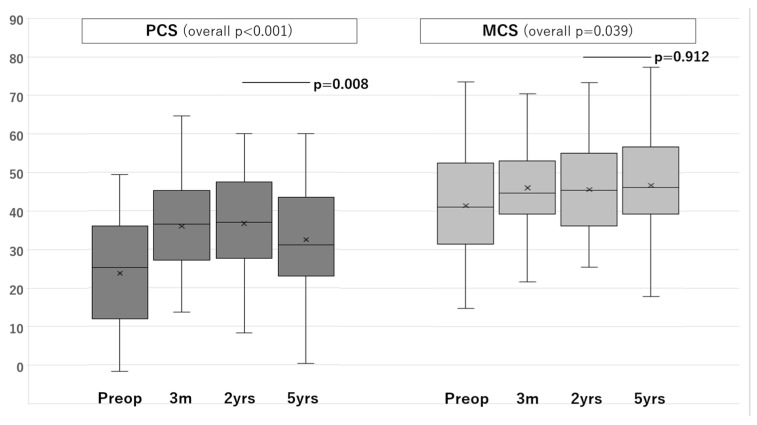
Changes in the average MCS and PCS scores after surgery.

There were significant changes in PCS and MCS scores after surgery (PCS, *p* < 0.001; MCS, *p* = 0.039, Figure 1). There was a significant increase in the 2-year-postoperative PCS score compared to in the preoperative score (*p* < 0.001); however, the 5-year-postoperative score was significantly lower than the 2-year-postoperative score (*p* = 0.008). Meanwhile, there was a significant increase in the 2-year-postoperative MCS score compared to that in the preoperative score (*p* < 0.001), and the score did not differ significantly between 2 and 5 years postoperatively (*p* = 0.912). Spearman’s rank correlation coefficient showed no significant correlation between the change in PCS and MCS scores and MRI findings (*p* = 0.422 and 0.217, respectively).

#### 3.2.2. Individual Change in PCS and MCS Scores

PCS and MCS scores deteriorated in 42 and 21 patients, respectively, between 2 and 5 years postoperatively (Table 2). The number of patients with deterioration was significantly higher in the PCS than in the MCS scores (*p* = 0.001).

### 3.3. Sub-Analysis

#### 3.3.1. Comparison of the Change in SF-36 Subdomain Scores

Based on the results of the main analysis, a sub-analysis of PCS was performed. Comparing the change in SF-36 domain scores after 2 years between the patients with and without deterioration in PCS scores, the patients with deterioration showed an association with significantly larger negative changes in all domains except SF and MH than that observed in those without deterioration (Table 3). The difference was largest in PF, followed by RP and BP.

#### 3.3.2. Predictive Factors for the Deterioration in PCS Scores

**Figure 2:**
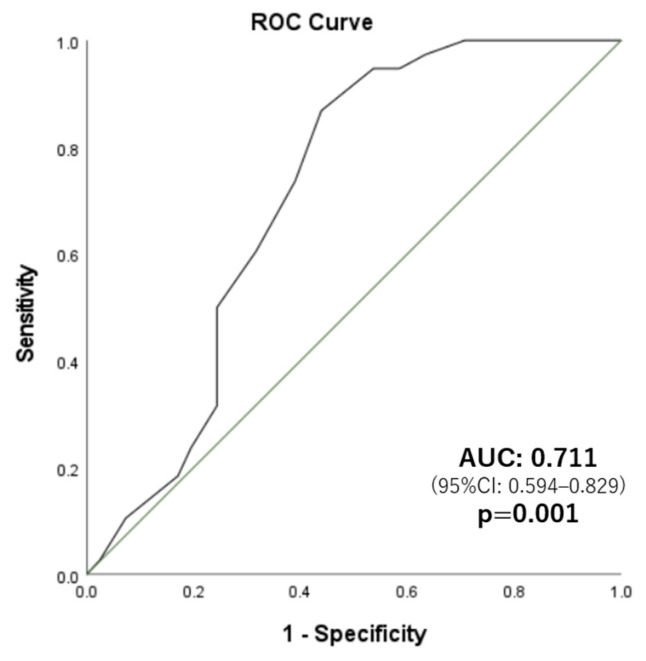
ROC analysis of cJOA score at 2 years postoperatively to predict PCS deterioration 2 years postoperatively.

Multivariate logistic regression analysis demonstrated that the cJOA score at 2 years postoperatively was significantly associated with the deterioration of PCS after 2 years of follow-up, independent of age, sex, and PCS score at 2 years postoperatively (*p* = 0.008, Aor = 0.57, Table 4). In the ROC analysis used to predict PCS deterioration after 2 years postoperatively, the AUC of the cJOA score at 2 years postoperatively was 0.711 (Figure 2, 95% CI 0.594–0.829, *p* = 0.001). The optimal cutoff value was 13.0 (sensitivity, 86.8%; specificity, 56.1%). Therefore, patients with cJOA scores of <13.0 at 2 years postoperatively experienced deterioration of PCS scores more frequently than patients with cJOA scores of ≥13.

## 4. Discussion

The findings of the current study indicate that the mental QOL of patients who underwent surgery for CSM did not show deterioration after 2 years of follow-up. Meanwhile, physical QOL significantly deteriorated between 2 and 5 years postoperatively. Several changes in the scores of the SF-36 domains, especially in the PF, RP, and BP domains, are relevant to PCS deterioration. Furthermore, we found that a cJOA score of <13.0 points at 2 years postoperatively might predict the deterioration of PCS between 2 and 5 years postoperatively.

Evaluation of patient-reported outcomes (PROs) has become a standard and essential method for identifying treatment efficacy [24]. PROs can be divided into two types based on their design: general and disease-specific PROs. The SF-36 is one of the most popular general PROs that evaluates patients’ mental and physical QOL and can be applied to all patients, regardless of the disease. Recent studies related to the spine have also applied the SF-36 as a parameter of surgical outcomes [7,15,25,26,27,28]. Therefore, we applied it as a parameter of QOL in the current study.

In 2021, Ghogawala et al. performed a large-scale randomized controlled study of patients with CSM and concluded that cervical laminoplasty improved physical QOL significantly at both 1 year and 2 years postoperatively [7]. Additionally, other retrospective cohort studies have revealed that up to 60% and 45% of patients with CSM experienced a meaningful improvement in their physical and mental QOL, respectively, at 2 years after cervical laminoplasty [15]. All such studies could prove that cervical laminoplasty for patients with CSM can improve not only the neurological symptoms but also QOL in a short-term follow-up period. In addition to such evidence, the current study findings provide knowledge of the changes in QOL after surgery for CSM after a short follow-up period.

It has been reported that improvement in mental-health-related QOL is decided within 3 months of surgery for CSM, independent of the recovery after treatment for myelopathy, and that there was a low chance of improvement over the next 21 months [15]. Meanwhile, the current study revealed that mental QOL will not deteriorate after a short-term period. These findings indicate that mental QOL is relatively stable, and physicians can focus on the first 3 months to achieve and maintain improvement in mental QOL.

In terms of physical QOL, many studies have demonstrated that improvement will continue in the short-term period [7,15,24,25,26,27]. However, the current study findings indicate that physical QOL will deteriorate between the short- and mid-term periods after surgery. This indicated that the improvement of physical QOL might have peaked in the short-term period; 52% of the patients in our study exhibited a subsequent decline in physical QOL. Moreover, we found that a cJOA score of <13.0 points at mid-term follow-up might predict deterioration afterward. The postulated mechanism based on our results was that patients with moderate-to-severe residual neurological symptoms might recognize the disability in their daily setting, resulting in the deterioration of physical QOL.

The current study has clear clinical relevance, which will help surgeons in some aspects. Our study suggests that mental QOL could be stable during the first 3 months. Therefore, it can be maintained and improved if physicians focus on it within that time period. However, physical QOL improves in the short-term period, and half of the patients with CSM reported a decline in physical QOL thereafter. Therefore, physicians may have to continue to follow-up on patients with a cJOA score of <13.0 for more than 2 years. Furthermore, physicians could consider additional therapy, such as rehabilitation and/or exercises, especially for patients with a cJOA score of <13.0 during the short-term follow-up period.

This study has several limitations. First, the study’s retrospective nature makes it difficult to exclude bias, especially regarding the referral to a specific postoperative rehabilitation program and the surgical techniques utilized. Second, although we used the SF-36 PCS and MCS as parameters of QOL, QOL status should be analyzed in a multifaceted manner, and the current result should be validated by further studies that consider other aspects [28]. Third, reoperation and surgical complications were not included in the variables for multivariate analysis because the numbers were too low. Finally, to evaluate a consistent population with regard to the surgical method, we included only patients treated with laminoplasty. Other surgical methods, including posterior decompression and fusion and anterior cervical discectomy and fusion, should be validated. However, this is the first study to elucidate the change in the QOL of patients with CSM after short-term follow-up and identify the short-term follow-up finding that can be considered a predictor for subsequent deterioration.

## 5. Conclusions

The mental QOL of patients who underwent surgery for CSM did not deteriorate after a short-term follow-up. Meanwhile, more than half of the patients experienced deterioration in physical QOL after short-term periods. A cJOA score of >13.0 points at short-term follow-up potentially predicted the deterioration of physical QOL afterwards. The current results suggest that physicians must continue follow-up for patients with a cJOA score of <13.0 for over 2 years. Furthermore, additional intervention may be planned for patients with a cJOA score <13.0 at the short-term follow-up.

## Figures and Tables

**Table 1:** Patient demographics.

Variables	Values	*p*-Value
Total numbers (cases)	80	
Age (years)	64.0 ± 11.2	
Sex (female/male)	25/55	
MRI findings		
Number of stenosis levels		
Single-level stenosis	12 (15.0%)	
Double-level stenosis	48 (60.0%)	
Triple-level stenosis	20 (25.0%)	
Snake-eye appearance (+)	31 (38.8%)	
Complications		
C5 palsy	3 (3.8%)	
Surgical site infection	1 (1.3%)	
Reoperation within 5 years	2 (2.6%)	
cJOA score		
Preoperative	9.7 ± 3.4	<0.001 *
3 months postop	13.3 ± 2.1	
2 years postop	13.5 ± 2.5	
5 years postop	13.1 ± 2.8	
Neck pain VAS		0.018 *
Preoperative	24.7 ± 29.0	
3 months postop	13.9 ± 20.4	
2 years postop	12.9 ± 21.1	
5 years postop	12.6 ± 20.8	
Arm pain VAS		0.045 *
Preoperative	31.4 ± 33.0	
3 months postop	20.0 ± 25.6	
2 years postop	17.0 ± 25.6	
5 years postop	16.6 ± 27.1	
Arm numbness		<0.001 *
Preoperative	58.6 ± 30.0	
3 months postop	33.9 ± 27.7	
2 years postop	33.4 ± 28.0	
5 years postop	34.1 ± 28.3	

* Repeated one-way analysis of variance. cJOA: cervical Japanese Orthopaedic Association; postop: postoperative; VAS: visual analog scale.

**Table 2:** Deterioration greater than the MCID between 2 and 5 years postoperatively.

	Improved or Stable	Deterioration	*p*-Value
SF-36 PCS	38	42	0.001 ^#^
SF-36 MCS	59	21	

“Deterioration” refers to patients who showed a positive change in the PCS or MCS score more than MCID (=4.0 points). ^#^: Chi-squared test; MCID: minimal clinically important difference; SF-36: Short Form-36; PCS: physical component summary; MCS: mental component summary.

**Table 3:** Univariate comparison of the change in SF-36 domain scores after 2 years between patients with and without PCS deterioration.

Domains	Without Deterioration	Deterioration	*p*-Value
Physical functioning	6.6 ± 12.0	−19.4 ± 19.5	<0.001
Role physical	4.4 ± 27.1	−17.4 ± 27.3	<0.001
Bodily pain	4.6 ± 14.6	−12.4 ± 23.8	<0.001
General health perceptions	0.8 ± 12.1	−5.9 ± 12.2	0.015
Vitality	1.8 ± 19.0	−7.6 ± 15.6	0.018
Social functioning	7.6 ± 20.0	−3.0 ± 27.9	0.058
Role emotional	7.7 ± 24.2	−8.9 ± 31.4	0.011
Mental health	0.7 ± 16.9	−4.2 ± 18.3	0.221

SF-36: Short Form-36; PCS: physical component summary.

**Table 4:** Multivariate logistic regression analysis of factors associated with PCS deterioration 2 years postoperatively.

Explanatory Variables	Reference	Adjusted OR	*p*-Value	95% CI
Age	Continuous	1.05	0.131	0.98–1.11
Sex (male)	Female	1.05	0.937	0.36–3.08
cJOA at 2 ys postop	Continuous	0.57	0.008	0.38–0.86
PCS at 2 ys postop	Continuous	1.07	0.052	0.99–1.14

cJOA: cervical Japanese Orthopaedic Association, PCS: physical component summary, 2 ys: 2 years, postoperative: postoperative.

## Data Availability

The datasets generated and/or analyzed during the current study are available from the corresponding author on reasonable request.
